# Oncogene and therapeutic target analyses in atypical fibroxanthomas and pleomorphic dermal sarcomas

**DOI:** 10.18632/oncotarget.7845

**Published:** 2016-03-02

**Authors:** Doris Helbig, Michaela Angelika Ihle, Katharina Pütz, Iliana Tantcheva-Poor, Cornelia Mauch, Reinhard Büttner, Alexander Quaas

**Affiliations:** ^1^ Department of Dermatology, University Hospital Cologne, Cologne, Germany; ^2^ Institute of Pathology, University Hospital Cologne, Cologne, Germany

**Keywords:** atypical fibroxanthoma, CDK4, CCND1, pleomorphic dermal sarcoma, TP53

## Abstract

**Background:**

Until now, almost nothing is known about the tumorigenesis of atypical fibroxanthoma (AFX) and pleomorphic dermal sarcoma (PDS). Our hypothesis is that AFX is the non-infiltrating precursor lesion of PDS.

**Materials and Methods:**

We performed the world-wide most comprehensive immunohistochemical and mutational analysis in well-defined AFX (n=5) and PDS (n=5).

**Results:**

In NGS-based mutation analyses of selected regions by a 17 hotspot gene panel of 102 amplicons we could detect *TP53* mutations in all PDS as well as in the only analyzed AFX and PDS of the same patient. Besides, we detected mutations in the *CDKN2A, HRAS, KNSTRN* and *PIK3CA* genes.

Performing immunohistochemistry for CTNNB1, KIT, CDK4, c-MYC, CTLA-4, CCND1, EGFR, EPCAM, ERBB2, IMP3, INI-1, MKI67, MDM2, MET, p40, TP53, PD-L1 and SOX2 overexpression of TP53, CCND1 and CDK4 was seen in AFX as well as in PDS. IMP3 was upregulated in 2 AFX (weak staining) and 4 PDS (strong staining).

FISH analyses for the genes *FGFR1, FGFR2* and *FGFR3* revealed negative results in all tumors.

**Conclusions:**

UV-induced *TP53* mutations as well as CCND1/CDK4 changes seem to play essential roles in tumorigenesis of PDS. Furthermore, we found some more interesting mutated genes in other oncogene pathways (activating mutations of *HRAS* and *PIK3CA*). All AFX and PDS investigated immunohistochemically presented with similar oncogene expression profiles (TP53, CCND1, CDK4 overexpression) and the single case with an AFX and PDS showed complete identical *TP53 and PIK3CA* mutation profiles in both tumors. This reinforces our hypothesis that AFX is the non-infiltrating precursor lesion of PDS.

## INTRODUCTION

Atypical fibroxanthoma (AFX) and pleomorphic dermal sarcoma (PDS) are rare tumors that typically arise on sun-exposed skin in the head and neck region of elderly patients [1]. Histologically, AFX can be composed of pleomorphic, spindle or epithelioid cells arranged in a haphazard or storiform pattern. The term “pleomorphic dermal sarcoma” was introduced by Fletcher [2] and describes tumors having been referred to “cutaneous undifferentiated pleomorphic sarcomas” or “superficial malignant fibrous histiocytomas” in the past. These tumors present with a similar morphology to AFX, but in addition, show extensive invasion of deeper structures [1, 2]. Both, AFX and PDS are usually negative for cytokeratins, S100, CD34, and desmin [3].

The most important risk factor for AFX is, similar to cutaneous squamous cell carcinomas (cSCC), UV exposure. The link of UV dependency to PDS is less clear. AFX generally do not recur after complete excision. In contrast, PDS, have the potential for local recurrence, metastasis and disease-specific death and there are no standard effective treatments beyond surgery and radiation in metastasized stages [4–6].

Until now, there is considerable controversy regarding the question if AFX and PDS are related neoplasms arising from a common mesenchymal progenitor cell, may represent “two poles of the same disease” or represent separate entities [5],

Over the last years, NGS analyses in cSCC identified common gene alterations in *TP53*, *NOTCH1*, *RAS*, *CDKN2A*, *AJUBA*, *CASP8*, *FAT1*, *KMT2C (MLL3)*, *PIK3CA*, *SOX2* and *CCND1* [7, 8]. In contrary, there are just a few small studies which identified UV-signature *TP53* mutations in AFX (66.7-70%) [4, 5]. To the best of our knowledge, there are no studies investigating other oncogenes or therapeutic structures in AFX or PDS.

For that reason, we performed, based on our hypothesis that AFX is the non-infiltrating precursor lesion of PDS, comparing immunohistochemical, NGS as well as FISH analyses of several proteins/genes in well characterized AFX and PDS samples including one case with an initially diagnosed AFX and a PDS 3 years later to get insights of their possible evolution. Furthermore, we hoped to identify diagnostic or prognostic markers as well as target structures for therapies in advanced tumor stages.

## RESULTS

### Immunohistochemistry (IHC)

The results of all immunohistochemical stainings are summarized in Table 1.

**Table 1:** IHC Results (+ slightly positive, ++ moderately positive, +++ strongly positive, - negative, NT no tumor)

	CCND1	CDK4	c-MYC	CTLA-4	CTNNB1	EGFR	EPCAM	ERBB2	IMP3	KIT	INI-1	MKI67	MDM2	MET	p40	TP53	PD-L1	SOX2
**AFX**																		
**+**		2				1			1			4×3-5%						
**++**	1								1		5					2		
**+++**	3											1×20-25%				2		
**-**	1	3	5	5	5	4	5	5	3	5			5	5	5	1	5	5
**NT**																		
**PDS**												1×3-5%						
**+**	3		1	3								4×10-15%			1			
**++**									4		6	1×20-25%				3		
**+++**	2	1				1												
**-**	1	5	3	2	6	5	6	6	1	6			6	6	4	2	6	6
**NT**			1	1					1						1	1		

### Positive expression

**Figure 1:**
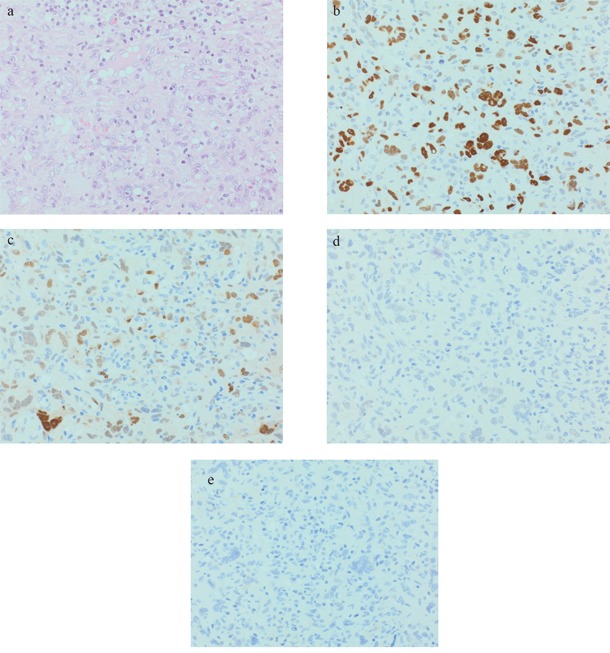
Representative AFX (case 4): a. 40xHE; b. 40xp53; c. 40xCyclin D1; d. 40xCDK4; e. 40xIMP3

**Figure 2:**
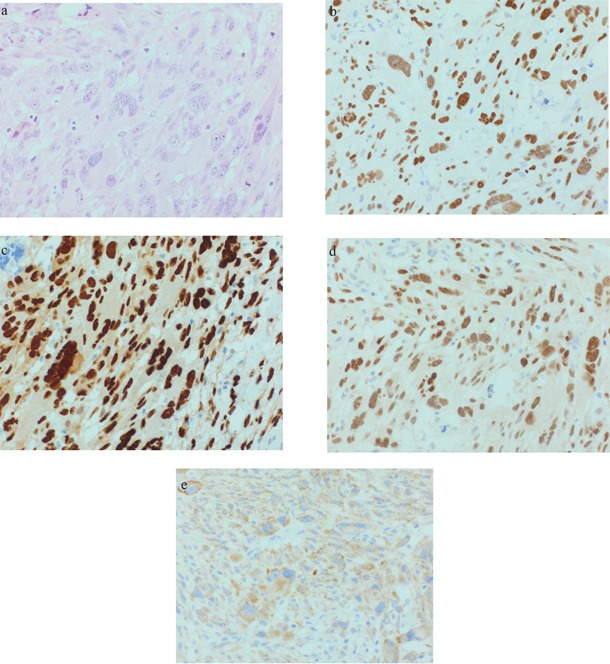
Representative PDS (case 8): a. 40xHE; b. 40xp53; c. 40xCyclin D1; d. 40xCDK4; e. 40xIMP3

In 9 analyzed tumors, TP53 was moderately to strongly expressed in 3 AFX and 2 PDS (see Figure 1 and 2b). CCND1 was moderately to strongly expressed in 3 AFX and 4 PDS. 2 AFX and 1 PDS with CCND1 expression were also strongly positive for CDK4 (see Figure 1 and 2c and 2d). IMP3 was upregulated in 1 AFX and 3 PDS (see Figure 1 and 2e). Case 5 showed strong TP53 and CCND1 expression in both tumor samples, CDK4 was negative and IMP3 was focally positive in the AFX and diffuse positive in the PDS sample. c-MYC was slightly positive in 1 PDS. For INI-1, no loss of expression could be found.

One case showed strong positivity for p40, TP53, CCND1 and EGFR. Although a pan-cytokeratin staining had been negative at the time of initial diagnosis, an additional CK5/6 staining was positive at present. Due to this, we had to revise the archival diagnosis of an AFX and diagnosed a SCC (not shown in the tables) and excluded the case from further analyses.

### Negative expression

EGFR was slightly expressed in 1 AFX and strongly expressed in 1 PDS corresponding to the usual skin expression of control samples. KIT was not expressed by any of the tumors. No expression of CTNNB1, CTLA4, EPCAM, ERBB2, MET, p40, PD-L1 and SOX2 could be detected in any of the tumors, PD-L1 was negative in tumor as well as stromal cells.

### Fluorescence *in-situ* hybridization (FISH)

We could not detect any *FGFR1* amplification in 8 of 10 tumors investigated. One AFX sample did not show any signal and one PDS sample exhibited too low tumor content to analyze 60 nuclei, our threshold for evaluating FISH analyses.

Moreover, we could not identify any *FGFR2* nor *FGFR3* translocation in our tumors.

### Next-generation-sequencing (NGS)

The results of all Next-Generation-Sequencing analyses are summarized in Table 2.

**Table 2:** Primer pairs and results of the NGS (n/a= not applicable= low DNA quality)

Gene	Exon	Codon	Mutation status	Freq. (%)	Interpretation
**BRAF**	11, 15	439-477, 582-620	Wild type		
**CDK4**	2	1-57	Wild type		
**CDKN2A**	1, 2	38-50, 136-152	**Case 6 (PDS):** EX1: c148C>T p.Q50* AND EX2: c.442G>A p.A148T	56.32 22.24	Truncated protein Described, function unknown [52]
**GNA11**	5	203-245	Wild type		
**GNAQ**	5	203-245	Wild type		
**HRAS**	2, 3, 4	1-37, 38-97, 98-146	**Case 6 (PDS):** EX2: c.37G>C p.G13R	19.34	Activating
**IDH1**	4	89-138	**Case 7 (PDS):** EX4: c379C>T p.P127S	18.02	Described, function unknown [53]
**KIT**	9, 11, 13, 17, 18	450-513, 550-591, 624-659, 784-824, 825-861	Wild type		
**KNSTRN**	1	1-38	**Case 6 (PDS):** EX1: c.71C>T p.S24F	29.04	Described [8]
**KRAS**	2-4	1-37, 41-92, 98-150	Wild type		
**NRAS**	2-4	1-37, 41-92, 98-150	Wild type		
**OXA1L**	1	1-77	Wild type		
**PDGFRA**	12, 14, 18	552-595, 632-667, 814-854	Wild type		
**PIK3CA**	9, 20	514-555, 980-1069	**Case 5 (AFX recurring as PDS, both tumors):** EX9: c.1624G>A p.E542K	35.99/48.07	Activating
			**Case 6 (PDS):** EX9: c.1633G>A p.E545K	36.43	Activating
**PTEN**	1-7	1-26, 27-55, 56-70, 71-84, 85-164, 165-211, 212-267, 268-342, 343-404	Wild type		
**RAC1**	2	13-35	Wild type		
			**Case 5 (AFX and PDS, both tumors):** EX6: c.672+1G>C AND EX8: c.818G>A p.R273H	19.19/24.6 36.47/47.68	Splice site base exchange Non-functional proteinneutral
			**Case 6 (PDS):** EX7: c.697C>T p.H233Y	14.22	neutral
**TP53**	5-9	126-186, 187-224, 225-261, 262-306, 307-331	**Case 7 (PDS):** EX6: c.637C>T p.R213*Case 8 (PDS)	24.98 n/a	Truncated protein
			**Case 9 (PDS)**: EX6: c.585_586CC>>TT p.R196* AND EX7: c.712T>A p.C238S	36.6 20.53	Truncated protein Non-functional protein
			**Case 10 (PDS):** EX5: c.490A>T p.K164* AND EX6: c.586C>T p.R196*	22.56 9.98	Truncated protein Truncated protein

### Mutated genes

By sequencing exons 5-8 of *TP53*, mutations could be detected in all 5 analyzable PDS (case 5, 6, 7, 9 and 10). In case 8, the tumor content was too low for mutation analyses (<10%). In the two PDS (case 6 and 10) which were immunohistochemically negative for TP53, *TP53* mutations could also be found (case 6 with a mutation without effect on the protein function; neutral, case 10 with a truncated protein, according to the IARC TP53 database, http://p53.iarc.fr/TP53GeneVariations.aspx).

**Figure 3:**
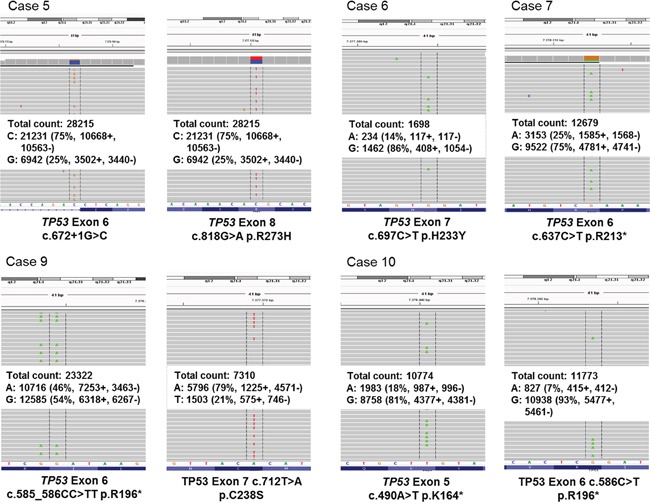
TP53 mutational analyses *TP53* exons 5-8 were analyzed by Next Generation Sequencing. Integrative genomic viewer visualization of all detected *TP53* mutations in AFX and PDS. In case 8, the tumor content was too low for mutation analyses (<10%).

Two of the 4 PDS (case 9 and 10) as well as the case with an AFX and PDS (case 5) had double-hit mutations in the *TP53* gene. Four *TP53* mutations took place at dipyrimidine sites being C to T transitions, case 9 carried a CC>TT tandem base substitution (see Figure 3).

Besides the *TP53* mutations, case 5 presented with an activating *PIK3CA* mutation and complete identical *TP53* and *PIK3CA* mutation pattern in both tumors. Case 6 had two mutations in the *CDKN2A*, one mutation in the *HRAS*, one in the *KNSTRN* and one activating mutation in the *PIK3CA* gene. Case 7 presented an *IDH1* mutation.

### Wild-type-genes

All other investigated genes (compare Table 2) did not show any mutation.

## DISCUSSION

*TP53* mutations seem to be essential in the development of PDS (driver mutation). Our mutation analyses using NGS could detect *TP53* mutations in all analyzable PDS as well as the AFX and PDS of one patient. The majority of our *TP53* mutated tumors are associated with an immunohistochemical TP53 expression. However, some TP53 mutated cases were immunohistochemically negative. This discrepancy has been reported by other investigators especially in the case of nonsense, splice and null mutations [9, 10]. The majority of the detected TP53 substitutions demonstrated characteristic UV-induced *TP53* mutations taking place at dipyrimidine sites being C to T transitions. This is in accordance with previous studies in UV-induced cSCC and basal cell carcinomas (BCC) [11]. All PDS investigated were located in UV-exposed localizations. Interestingly, 3 of 5 PDS (including the case with an AFX and PDS) had double-hit mutations in the *TP53* gene not showing the typical C to T transitions in the second hit.

The tumor-suppressor protein TP53 is a transcriptional activator which is involved in cell-cycle control, DNA repair and apoptosis in response to a variety of stimuli. Loss of p53 functionality is known to result in impaired growth arrest and inappropriate cell survival explaining the fact that *TP53* is the most frequently mutated gene in a wide range of human cancers [12–14].

Furthermore, we detected additional mutations in the *CDKN2A, KNSTRN, HRAS* and *PIK3CA* genes in a single PDS. Another activating mutation of *PIK3CA* gene was found in the recurring tumor. Phosphatidylinositol 3-kinases (PI3Ks) are lipid kinases that regulate signaling pathways important in the tumorigenesis of solid and mesenchymal tumors [15–19]. Preclinical and clinical studies in patients with breast, cervical, endometrial, and ovarian cancer treated with PI3K/AKT/mTOR inhibitors could demonstrate a higher response rate in patients with activating *PIK3CA* mutation than patients without mutation [20–22]. A subset of dermal sarcomas with identified activating *PIK3CA* mutation might therefore respond to a treatment with PI3K inhibitors.

*CDKN2A* mutations have been frequently detected in other UV-induced tumors as well as in few sarcomas [8, 23–25]. The double-hit mutation in *CDKN2A* that we detected in one case (EX1: c148C>T p.Q50* and EX2: c.442G>A p.A148T) leads to a non-functional protein. We speculate that this mutation leads to a lacking inhibition of the CCND1–CDK4/6 complexes.

The GTPase HRAS is involved in regulating cell division in response to growth factor stimulation. Our detected activating *HRAS* mutation has the capacity to a ligand-independent cell growth/proliferation [26, 27].

The detected *KNSTRN* mutation in one of our tumors is located in a UV-signature hotspot [28, 29]. *Kinastrin* (kinetochore-localized astrin/SPAG5 binding protein (*KNSTRN*)) encodes a kinetochore-associated protein that is an essential component of the mitotic spindle and is required for faithful chromosomal segregation during mitosis. It is expressed in a broad range of tissues, including skin and seems to promote genomic stability [28, 29].

Our case 7 harbored an additional *isocitrate dehydrogenase* (*IDH)1* mutation (EX4: c379C>T p.P127S) with unknown function. Dysfunctional IDH leads to reduced production of α-ketoglutarate and NADH and increased production of 2-hydroxyglutarate, an oncometabolite. *IDH1* mutations were originally reported as frequent events in glioblastomas as well as acute myeloid leukemia [30–32].

Another new finding of our investigations is the immunohistochemical overexpression of CCND1 and/or CDK4 in 9 of 11 tumors. Therefore, deregulation of the CCND1/CDK4/6/RB1 pathway or mutations, amplifications and overexpression of the *CCND1* gene are frequently observed in different malignomas including UV-related cSCC. In this context, CCND1 overexpression usually occurs early during tumorigenesis and the increased levels of CCND1 seem to result more frequently from a defective regulation at a post-translational level, rather than from a somatic mutation or rearrangement in the *CCND1* gene [33–36].

Our findings of CCND1 and/or CDK4 allow us the suggestion that the selective CDK4/6 inhibitor Palbociclib (IBRANCE®, Pfizer Inc.) may be a promising treatment option in unresectable or metastasized tumor stages. Palbociclib (= PD0332991) has been approved in 2015 by the FDA for the treatment of metastasized ER+, ERBB2-negative breast cancer in postmenopausal women in combination with Letrozole [37]. Due to the fact that patients benefit from this therapy regardless of the biomarker status Palbociclib was approved for an unselected population [37].

Moreover, we saw an insulin-like growth factor II (IGF-II) mRNA binding protein 3 (IMP3) immunohistochemical overexpression in 2 of our 5 AFX and 4 of 5 analyzable PDS. There was a focally positivity in the AFX and a diffuse positivity in the PDS of the same patient. IMP3 is a member of the insulin-like growth factor II mRNA binding protein family [38]. It has been shown to be involved in tumorigenesis of certain malignant neoplasms and is correlated with worse prognosis in some carcinomas [39–45]. In regard to sarcomas, cytoplasmic IMP3 expression of varying intensity was detected in 52-100% of cutaneous leiomyosarcomas in contrast to typical leiomyomas [46]. Larger studies need to be performed to investigate if IMP3 staining may be useful for discriminating AFX from PDS in routine praxis or could be used as an indicator of a more aggressive clinical behavior in AFX (AFX with advanced tendency to PDS).

In conclusion, UV-induced mutations, especially *TP53* mutations and CCND1/CDK4 alterations seem to be essential in the development of PDS. Furthermore, we found some more interesting mutated genes in other pathways (activated mutations of *HRAS, PIK3CA* and *CDKN2A*). In unresectable or metastasizing tumor stages CDK4/6 inhibitors (Palbociclib) or PI3K inhibitors (Pazopanib) seem to be promising treatment options. A targeted therapy could further be useful to size down the tumor before operation or in the case of inoperable patients.

The detection of *TP53* mutations in all tumors as well as a complete identical exon mutation profile in the AFX and PDS of the same patient seems to emphasize our hypothesis that AFX is an UV-induced non-infiltrating precursor of PDS. IMP3 has probably the potential to discriminate AFX from PDS or could be used as an indicator of a more aggressive clinical behavior in AFX.

## MATERIALS AND METHODS

### Patient characteristics and tumor material

11 cases (5x AFX and 6x PDS) were included into this study (see Table 3). Case 5 consisted of two tumor samples: primary: an AFX, which had been completely excised, and secondly: a PDS three years after the initial diagnosis.

**Table 3:** Patient and Tumor Characteristics

	AFX (cases 1-5)	PDS (cases 5-10)	AFX and PDS of the same patient (case 5)
**Male**	4	6	1
**Female**	1	0	0
**Age range; median (years)**	75-84; 81	66-89; 82.5	84 (AFX) – 87 (PDS)
**Tumor localization** - **Capillitium** - **Thigh**	4 1	5	1

**Table 4:** Antibodies used for immunohistochemistry

Antibody specificity	Species and type	Clone and Catalog No.	Source	Conditions
**CCND1**	Rabbit; IgG	SP4; 241R-16	Cellmarque	EDTA; 1:25
**CDK4**	Mouse; IgG1	DCS-31; AH20202	Invitrogen	EDTA; 1:100
**c-MYC**	Rabbit; IgG	Y69; 1472-1	Epitomics	Citrate; 1:100
**CTLA-4**	Mouse; IgG1	F-8; sc-376016	Santa Cruz	EDTA; 1:400
**CTNNB1**	Rabbit; IgG	Polyclonal; RB9035-p	ThermoScientific	Citrate; 1:400
**EGFR**	Mouse; IgG1	2-18C9; K1492	Dako pharm Dx	Ready-to-use
**EPCAM**	Mouse; IgG1, K	MOC-31; 248M-16	Cellmarque	Citrate; 1:400
**ERBB2**	Rabbit; IgG	4B5; 52783600	Ventana/Roche	Ready-to-use
**IMP3**	Mouse; IgG2a	69.1; M3626	Dako	EDTA; 1:100
**INI-1**	Mouse; IgG2a	MRQ-27; 272M-16	Cellmarque	EDTA; 1:100
**KIT**	Rabbit; IgG1	Polyclonal; A4502	Dako	Citrate; 1:400
**MKI67**	Rabbit; IgG	SP6; 275R-16	Cellmarque	EDTA; 1:100
**MDM2**	Mouse; IgG2b	IF2; 18-2403	Invitrogen	EDTA; 1:100
**MET**	Rabbit; IgG	SP44; 790-4430	Ventana/Roche	Ready-to-use
**p40**	Rabbit; IgG	Polyclonal; ACI3030B	Zytomed	EDTA; 1:50
**TP53**	Mouse; IgG2b	DO-7; M7001	Dako	Citrate; 1:800
**PD-L1**	Rabbit; IgG	28-8; Ab205921	Abcam/Dako	EDTA; 1:100
**SOX2**	Rabbit; IgG	SP76; 371R-16	Cellmarque	EDTA; 1:50

All tumors were selected due to their “typical” morphology, AFX had to be well-defined to the dermis in contrast to the PDS which had to present infiltration of the subcutis. Immunohistochemical stainings performed at the time of diagnosis had to include at least one cytokeratin (such as CK 5/6 or pan-cytokeratin), two melanocytic (such as S100 and melan A, HMB45) and vascular markers (such as CD31, CD34, podoplanin) to exclude other entities.

### Tissue microarray (TMA)

Two tissue cores from different areas of each tumor were punched out and transferred in a TMA recipient block. TMA construction was performed as described earlier [47]. In brief, tissue cylinders with a diameter of 1.2 mm each were punched from selected tumor tissue blocks using a homemade semi-automated precision instrument and brought into empty recipient paraffin blocks. Four μm sections of the resulting TMA blocks were transferred to an adhesive coated slide system (Instrumedics Inc., Hackensack, NJ). Consecutive sections were used for fluorescence *in-situ* hybridization (FISH) and immunohistochemistry.

### Immunohistochemistry

Immunohistochemical stainings were performed using the BOND MAX from Leica (Leica, Germany) according to the protocol of the manufacturers. For details of all antibodies see Table 4. All immunostainings were scored independently by one dermatopathologist (D.H.) and one pathologist (A.Q.).

### FISH

### *FGFR1* amplification analysis

A Spectrum green-labeled FGFR1-probe (8p11.23-p11.22) was used together with a Spectrum orange-labeled CEN8 probe (ZytoLight® SPEC FGFR1/CEN 8 Dual Color Probe) for *FGFR1* amplification analysis as described before [48, 49].

### *FGFR2* and *FGFR3* translocation analysis

Translocations affecting the *FGFR2* gene have been investigated using *FGFR2* break-apart FISH probe mix (ZytoVision, Germany), *FGFR3* gene have been investigated using the ZytoLight® SPEC FGFR3 Dual Color Break Apart Probe (ZytoVision, Germany) designed to detect rearrangements involving the chromosomal region 4p16.3 - for details compare literature [50].

### Next-generation-sequencing (NGS)

Tumors were diagnosed by an experienced pathologist and dermatopathologist (A.Q. and D.H.) and tumor content was defined.

All samples were fixed in neutral-buffered formalin prior to paraffin embedding (FFPE-samples). On a haematoxylin-eosin stained slide tumor areas were selected by a pathologist and dermatopathologist (A.Q. and D.H.) and DNA was extracted from three corresponding unstained 10 μm thick slides by manual micro-dissection as described before [51]. The DNA was isolated by semi-automated extraction with the Maxwell^®^ 16 FFPE Plus Tissue LEV DNA Purification Kit (Maxwell^®^ 16, Promega). DNA extracts were quantified by qPCR.

Targeted next generation sequencing (NGS) was performed with an in-house specified, customized primer panel of 17 different genes (*BRAF* exons 11, 15; *CDK4* exon 2; *CDKN2A* exons 1, 2; *GNA11* exon 5; *GNAQ* exon 5; *HRAS* exons 2-4; *IDH1* exon 4; *KIT* exons 9, 11, 13, 17, 18; *KNSTRN* exon 1; *KRAS* exons 2-4; *NRAS* exons 2-4; *OXA1L* exon 1, *PDGFRA* exons 12, 14, 18; *PIK3CA* exons 9, 20; *PTEN* exons 1-7, *RAC1* exon 2, *TP53* exons 5-9). Library preparation was performed with the GeneRead™ DNAseq targeted Panels V2 Kit from Qiagen.

Sequencing was done on an Illumina MiSeq benchtop sequencer (Illumina, San Diego, USA). Results were visualized in the Integrative Genomics Viewer (IGV) [6] and manually analyzed.

Quality criteria for mutation calling were a coverage >200, an allelic frequency of 5% mutated allele in a background of wildtype alleles and a tumor content of >10%. Samples that did not fulfill these criteria were excluded from this study and determined as not analyzable.

NGS was performed in 6 PDS (cases 5-10) and one AFX (Case 5: the patient who developed an AFX and PDS).
